# Association of Long-Term Diet Quality with Hippocampal Volume: Longitudinal Cohort Study

**DOI:** 10.1016/j.amjmed.2018.07.001

**Published:** 2018-11

**Authors:** Tasnime Akbaraly, Claire Sexton, Enikő Zsoldos, Abda Mahmood, Nicola Filippini, Clarisse Kerleau, Jean-Michel Verdier, Marianna Virtanen, Audrey Gabelle, Klaus P. Ebmeier, Mika Kivimaki

**Affiliations:** a MMDN, University of Montpellier, EPHE, INSERM U1198, PSL Research University, Montpellier, France; b Department of Epidemiology and Public Health, University College London, UK; c Department of Psychiatry & Autism Resources Centre, Hospital and University Research Center of Montpellier, France; d FMRIB Centre, Nuffield Department of Clinical Neurosciences, University of Oxford, UK; e Neurobiology of Ageing Group, Department of Psychiatry, University of Oxford, UK; f Department of Public Health and Caring Sciences, Uppsala University, Sweden; g Memory Resources and Research Center for Alzheimer's Disease and Related Disorders, Department of Neurology, Gui de Chauliac Hospital, Montpellier, University of Montpellier, INSERM U1183, France

**Keywords:** Alternative Healthy Eating Index, Dietary indices, Hippocampal volume, Older adults, Prospective study

## Abstract

**BACKGROUND:**

Diet quality is associated with brain aging outcomes. However, few studies have explored in humans the brain structures potentially affected by long-term diet quality. We examined whether cumulative average of the Alternative Healthy Eating Index 2010 (AHEI-2010) score during adult life (an 11-year exposure period) is associated with hippocampal volume.

**METHODS:**

Analyses were based on data from 459 participants of the Whitehall II imaging sub-study (mean age [standard deviation] (SD) = 59.6 [5.3] years in 2002-2004, 19.2% women). Multimodal magnetic resonance imaging examination was performed at the end of follow-up (2015-2016). Structural images were acquired using a high-resolution 3-dimensional T1-weighted sequence and processed with Functional Magnetic Resonance Imaging of the Brain Software Library (FSL) tools. An automated model-based segmentation and registration tool was applied to extract hippocampal volumes.

**RESULTS:**

Higher AHEI-2010 cumulative average score (reflecting long-term healthy diet quality) was associated with a larger total hippocampal volume. For each 1 SD (SD = 8.7 points) increment in AHEI-2010 score, an increase of 92.5 mm^3^ (standard error = 42.0 mm^3^) in total hippocampal volume was observed. This association was independent of sociodemographic factors, smoking habits, physical activity, cardiometabolic health factors, cognitive impairment, and depressive symptoms, and was more pronounced in the left hippocampus than in the right hippocampus. Of the AHEI-2010 components, no or light alcohol consumption was independently associated with larger hippocampal volume.

**CONCLUSIONS:**

Higher long-term AHEI-2010 scores were associated with larger hippocampal volume. Accounting for the importance of hippocampal structures in several neuropsychiatric diseases, our findings reaffirm the need to consider adherence to healthy dietary recommendation in multi-interventional programs to promote healthy brain aging.

**Clinical Significance**

• Healthy diet is associated with reduced risk of depression and brain aging outcomes and periodontium, to the complete loss of teeth.
• Few studies have explored brain structures in humans potentially affected by diet.
• None of them examined the impact of long term diet on hippocampus.
• Long-term adherence to healthy diet was associated with larger hippocampal volumes
• The key component associated with larger hippocampus volume is low alcohol intake

Alt-text: Unlabelled box

## Introduction

Findings from cohort studies suggest that healthy diet (ie, a diet rich in anti-oxidants and anti-inflammatory compounds^1^ that improve insulin sensitivity and endothelial function) may also prevent depression and delay cognitive decline.^2^, ^3^, ^4^ In parallel, research investigating mechanisms by which overall diet might exert its protective effects on the brain is starting to emerge. Indeed, rodent models have shown that a diet rich in saturated fat, trans fat, and sugar adversely affects learning and memory performances that rely on the integrity of the hippocampus.^5^, ^6^ However, few studies have directly explored brain structures in humans that are potentially affected by diet or the extent to which healthy diets may protect from impairments in hippocampal structure or functions.^7^

Given the central role of the hippocampus in several neuropsychiatric diseases such as depression^8^, ^9^ and cognitive impairment,^10^ the hypothesis that a healthy diet may protect against these conditions by exerting positive effects on hippocampal structure is plausible. However, to establish an association between overall diet and specific brain structure, studies of humans that assess long-term dietary behaviors and measures of regional brain structure volumes are needed. To our knowledge, only 1 study has examined this issue, finding an independent association between unhealthy dietary patterns and smaller left hippocampal volumes in 255 Australian older adults.^11^

Our aim was to determine whether long-term adherence to healthy diet guidelines, based on recommendations in the Alternative Healthy Eating Index 2010 (AHEI-2010)^12^ during adult life is associated with subsequent hippocampal volume in a much larger sample of community-dwelling adults, the Whitehall II imaging sub-study. AHEI-2010 assessment performed 3 times over 11 years of follow-up (1991-1993–2003-2004), to predict brain structure in 2015-2016.

## Methods

Five hundred and fifty people were randomly selected for the current Whitehall II imaging sub-study (2012-2015)^13^ from the Whitehall II cohort study,^14^ a large-scale prospective cohort study of 10,308 civil servants recruited from 1985-1988 (phase 1). Since phase 1, follow-up examinations have taken place approximately every 5 years (phase 3: 1991-1993, phase 5: 1997-1999, phase 7: 2003-2004, phase 9: 2007-2009, phase 11: 2011-2012). This study was approved as part of a larger study (Predicting MRI abnormalities with longitudinal data of the Whitehall II sub-study; MSD/IDREC/C1/2011/71) by the University of Oxford's Medical Sciences Interdivisional Research Ethics Committee (reference: MSD/ IDREC/C1/2011/71).

### Assessment of Dietary Intake

Dietary intake was assessed from 1991-1993, 1997-1999, and 2003-2004, with the use of a semi-quantitative food frequency questionnaire (FFQ) with 127 food items, as described previously. Nutrient values were calculated using a computerized system developed for the Whitehall II dietary data, detailed in the online Appendix (Text 1). AHEI-2010 is based on 11 components: 6 components for which the highest intakes are supposed to be ideal: vegetables, fruit, whole grains, nuts and legumes, long chain omega-3 fats, and polyunsaturated fatty acids; and 4 components for which avoidance or lowest intake are supposed to be ideal: sugar-sweetened drinks and fruit juice, red and processed meat, trans fat, and sodium.^12^ In the original score, moderate alcohol intake was considered to be ideal; however, for brain related outcomes latest evidence supports to recommend avoidance or low consumption of alcohol rather than moderate consumption.^15^, ^16^ Scoring criteria for AHEI-2010 and its distribution are described in the online supplementary material (Supplementary Table 1).

We computed the AHEI-2010 scores from FFQ administered in phase 3 (1991-1993), phase 5 (1997-1999) and phase 7 (2002-2004). To reduce measurement errors and to represent long-term dietary intake, we calculated the cumulative average of AHEI-2010 over an 11-years exposure period. To analyze the association of change in AHEI score with hippocampal volumes, scores of AHEI at phase 3 and phase 7 were categorized as high or low according to the median value of AHEI-2010 score at phase 3 (60 points). Four categories were defined: participants who maintained a high score (both phase 3 and phase 7 scores ≥60.0), those who maintained a low score (both phase 3 and phase 7 scores <60.0), and participants who improved their AHEI score (phase 3 score <60.0 and phase 7 score ≥60.0) and those whose score decreased (phase 3 score ≥60.0 points and phase 7 score <60.0 points).

### Magnetic Resonance Imaging Acquisition and Processing and Assessment of Hippocampal Volume in 2015-2016

Multimodal magnetic resonance imaging (MRI) scans were acquired at the Oxford Centre for Functional MRI of the Brain (FMRIB Centre) using a 3-tesla MRI scanner (MAGNETOM Verio; Siemens Healthineers, Erlangen, Germany) with a 32-channel head coil. Details of the imaging protocol and the analysis pipelines have been published previously.^17^ In short, structural images were acquired using a high-resolution 3-dimensional T1-weighted sequence: repetition time = 2530 ms, echo time = 7.37 ms, flip angle = 78 degrees, field of view = 256 mm, and voxel dimensions = 1.0 mm isotropic. MRI data processing and analysis was performed using FSL tools (FMRIB Software Library; FMRIB, Oxford, UK). Structural, T1-weighted images were processed using fsl_anat (FMRIB). Details on brain tissue segmentation and hippocampal volume extractions and normalizations are detailed in the footnotes of Table 2.^17^, ^18^

### Statistical Analysis

First, linear regression models were performed to estimate the association between AHEI-2010 score and hippocampal volumes. The overall AHEI-2010 score was analyzed as a continuous standardized variable by using *z* score, and models were adjusted for age, sex, and total energy intake (model 1), then further adjusted for ethnicity, occupational position,^14^ smoking status, physical activity,^19^ health status factors (including coronary heart diseases, dyslipidemia, type II diabetes, body mass index [BMI] and hypertension) (model 2), and finally additionally adjusted for cognitive impairment^20^ and depressive symptoms^21^ (model 3). Assessment (2002-2004) and categorization of the covariates are detailed in the footnotes of Table 1. We performed supplementary analyses to assess 1) whether the significant associations between AHEI-2010 and hippocampal volumes remained in participants without cardiometabolic disease, cognitive impairment, and depressive symptoms and 2) whether the 11-year change in AHEI-2010 score was associated with subsequent hippocampal volumes.

**Table 1:** Characteristics of the 459 Participants of the Whitehall II Imaging Sub-Study

Characteristics of Participants from 2002-2004*		Description of Whitehall II Imaging Sub-Study Participants		Distribution of AHEI-2010†	
Sociodemographic Factors		N	% or mean (SD)	ρ ormean (SD)	*P*‡
Age, years		459	59.6 (5.3)	0.14	.005
Sex	Men	371	80.8	54.9 (8.3)	.23
	Women	88		57.9 (9.5)	
Ethnicity	White	432	94.1	54.9 (8.4)	.0002
	Nonwhite	27		63.7 (10.5)	
Socioeconomic status	Low/mid	187	41.1	55.7 (8.9)	.45
	High	272		55.1 (8.6)	
**Health behavior factors**					
Smoking status	Non/former	436	94.8	55.8 (8.6)	.0004
	Current	23		48.5 (8.2)	
Physical activity	Inactive /moderatelyactive	181	23.7	54.8 (9.1)	.21
	Active	278		55.9 (8.5)	
Total energy intake (kcal/d)		459	2190 (557)	-0.062	.18
**Health status factors**					
Antecedent of CHD	Yes	18	3.9	58.9 (7.3)	.35
	No	441		55.3 (8.8)	
Type II diabetes	Yes	38	8.2	57.2 (9.5)	.35
	No	421		55.3 (8.7)	
Hypertension	Yes	138	30.2	56.0 (8.6)	.41
	No	321		55.2 (8.8)	
BMI kg/m²		459	26.4 (3.8)	-0.077	.10
Dyslipidemia	Yes	74	16.2	55.2 (8.0)	.75
	No	385		55.5 (8.9)	
Cognitive impairment	Yes	41	9.2	55.7 (9.9)	.85
	No	403		55.4 (8.6)	
Depressive symptoms	Yes	63	14.7	53.5 (8.1)	.08
	No	366		55.6 (8.8)	

BMI = body mass index; CHD = coronary heart disease; SD = standard deviation.

⁎ Assessment of covariates: When possible covariates were obtained from the 2002-2004 study phase. Sociodemographic factors included sex, age, ethnicity (white/nonwhite) and occupational position, categorized into 3 groups: high (administrative), intermediate (professional or executive) and low (clerical or support). This measure is a comprehensive marker of socioeconomic circumstances in the Whitehall II study being related to education, salary, social status and level of responsibility at work.^14^ Health behaviors consisted of smoking status (self-reported and classified as “current smoker” or “noncurrent smoker” [including former smokers]), total energy intake (estimated from a food frequency questionnaire), and physical activity, assessed by a questionnaire including 20 items on frequency and duration of participation in different physical activities (eg, walking, cycling, and sports) that were used to compute hours per week at each intensity level. Participants were classified as “active” (>2.5 hours per week of moderate physical activity or >1 hour per week of vigorous physical activity), “inactive” (<1 hour per week of moderate physical activity and <1 hour per week of vigorous physical activity), or “moderately active” (if neither active nor inactive).^19^ Health status factors included prevalent CHD (denoted by clinically verified nonfatal myocardial infarction or definite angina); hypertension (defined by systolic/diastolic blood pressure ≥140 /90 mm Hg, respectively, or use of antihypertensive drugs); BMI; type II diabetes (diagnosed according to the World Health Organization definition); dyslipidemia (defined by high-density lipoprotein cholesterol <1.04 mmol/l and <1.29 mmol/l in men and women, respectively, or use of lipid-lowering drugs); cognitive impairment defined by a score ≤27 in the Mini-Mental State Exam^20^; and depressive symptoms defined by a score in the Center for Epidemiologic Studies Depression Scale^21^ ≥16, or being under antidepressant treatment. When there was a missing value for a covariate assessed at phase 7 (2002-2004), we imputed the value available at previous phases. We have done this for all covariates at exception of cognitive impairment and depressive symptoms.

† Cumulative average of Alternative Healthy Eating Index 2010 score over the 11-year exposure period (1991-1993–2002-2004).

‡ Means (m ± SD) of cumulative average of Alternative Healthy Eating Index 2010 score according to characteristics of participants were compared using the Student *t* test for categorized variables and Pearson correlation coefficients (ρ) were computed for quantitative variables.

Second, linear regression models described above were repeated for each AHEI-2010 component to identify the key components of the AHEI-2010 associated with hippocampal volumes. To further examine the contribution of each AHEI-2010 components to the overall AHEI-2010-hippocampal volumes association, we computed for each component (component i), a modified AHEI-2010 score based on the total AHEI-2010 score without the component i (modified AHEI-2010 score i = total AHEI-2010 score – score of the component i). All component scores and modified AHEI-2010 scores were standardized by using *z* scores. Analyses were conducted using SAS software, version 9.4 (SAS Institute, Cary, NC).

## Results

### Participants’ Descriptive Data

**Supplementary Figure 1:**
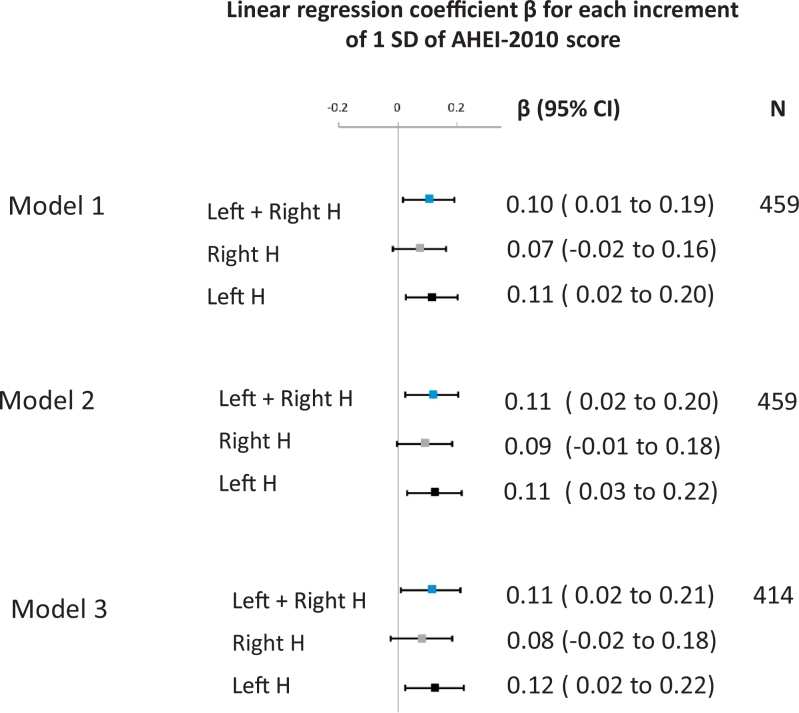
Flow chart diagram mapping the selection of participants.

Of the 550 Whitehall II imaging sub-study participants, 459 were included in the main analyses. The selection of participants is detailed in the online supplementary material (Supplementary Figure 1). Excluded participants and those included did not substantially differ in any of the reported characteristics (data available upon request). Characteristics of the 459 participants are presented in Table 1.

Distribution of cumulative average AHEI-2010 score according to the characteristics of participants is also detailed in Table 1. Means of AHEI-2010 score increased with age. A significantly lower mean AHEI-2010 score (ie, less healthy diet) was found in white participants compared with nonwhite participants and in smokers compared with former and nonsmokers. AHEI-2010 was inversely associated with BMI and tended to be lower in participants with depressive symptoms.

Distributions of hippocampal volumes (total, right, and left) as a function of participants’ characteristics are presented in Table 2. Advanced age was associated with lower hippocampal volumes. Participants with type II diabetes and those with hypertension were more likely to have lower hippocampal volumes. No significant differences in hippocampal volumes were observed for other baseline characteristics.

**Table 2:** Hippocampal Volumes According to Characteristics of Whitehall II Imaging Sub-study Participants

		Hippocampal Volumes*							
			Total		Right			Left	
Mean (SD)			**6839** (779)		**3468** (416)			**3371 (**433)	
**Characteristics**			ρ or Mean (SD)	*P*†	ρ or Mean (SD)		*P*†	ρ or Mean (SD)	*P*†
Age	Year		−0.31	<.001	−0.28	<.001		−0.29	<.001
Sex	Men		6839 (809)	.98	3470 (434)	.80		3369 (445)	.83
	Women		6838 (642)		3457 (331)			3380 (381)	
Ethnicity	White		6846 (660)	.40	3392 (314)	.33		3374 (435)	.56
	Nonwhite		6716 (660)		3472 (421)			3325 (403)	
Socioeconomic position	Low/mid		6783 (833)	.20	3444 (454)	.30		3339 (452)	.19
	High		6877 (739)		3484 (389)			3393 (419)	
Smoking status	Non/former		6849 (779)	.25	3469 (420)	.81		3380 (428)	.06
	Current		6660 (773)		3447 (346)			3213 (501)	
Physical activity	Inactive		6835 (851)	.97	3456 (440)	.80		3382 (472)	.97
	Moderately active		6822 (681)		3450 (375)			3369 (374)	
	Active		6845 (775)		3477 (417)			3369 (433)	
Total energy intake	kcal/d		0.009	.84	−0.025	.59		0.085	.37
Type II diabetes	No		6864 (787)	.02	3480 (423)	.007		3385 (434)	.02
	Yes		6552 (623)		3332 (297)			3220 (390)	
CHD	No		6847 (784)	.26	3472 (418)	.27		3375 (437)	.33
	Yes		6637 (605)		3361 (355)			3275 (315)	
Hypertension	No		6897 (795)	.01	3496 (427)	.02		3402 (438)	.02
	Yes		6702 (724)		3402 (383)			3300 (417)	
BMI	kg/m²		0.009	.83	−0.025	.58		0.04	.37
Dyslipidemia	No		6839 (800)	.97	3471 (427)	.65		3368 (440)	.74
	Yes		6836 (661)		3450 (352)			3386 (395)	
Cognitive impairment	No		6841 (788)	.63	3467 (421)	.75		3374 (437)	.57
	Yes		6779 (783)		3445 (397)			3334 (453)	
Depressive symptoms	No		6832 (770)	.90	3470 (416)	.75		3362 (425)	.60
	Yes		6846 (912)		3453 (473)			3393 (493)	
**Brain volumes**									
Total intracranial volumes	cm^3^		0.003	.95	−0.002	.96		0.007	.87
Total hippocampal volume	mm^3^		/	/	0.91	<.001		0.92	<.001
Right hippocampal volume	mm^3^		/	/	/	/		0.68	<.001

BMI = body mass index; CHD = coronary heart disease; SD = standard deviation.

⁎ MRI data processing and analysis used FSL tools (FMRIB Software Library, Oxford, UK). Structural, T1-weighted images were processed using fsl_anat (FMRIB). Brain tissues were segmented using FAST (FMRIB's Automated Segmentation Tool) that allows extracting measures of total gray matter, white matter, and cerebrospinal fluid, which were summed to calculate intracranial volume (ICV). FIRST (FMRIB),^17^ an automated model-based segmentation/registration tool, was applied to extract hippocampal volumes. Brain tissues and subcortical regions were visually inspected to ensure an accurate segmentation, and manually edited if required. Hippocampal volumes were normalized using a residual approach, which involves using a linear regression between the hippocampal volume and ICV to predict the ICV adjusted volumes.^18^ The formula: Voladj = vol – b × (ICV – mean ICV), where b is the regression coefficient of hippocampal volumes on ICV. All normalized hippocampal volumes and intracranial volumes were subsequently scaled to SD units by computing *z* scores.

† Student *t* test and analysis of variance for categorized variables and Pearson correlation coefficients (ρ) for quantitative variables.

### Long-Term Overall Diet Quality and Hippocampal Volume

**Figure 1:**
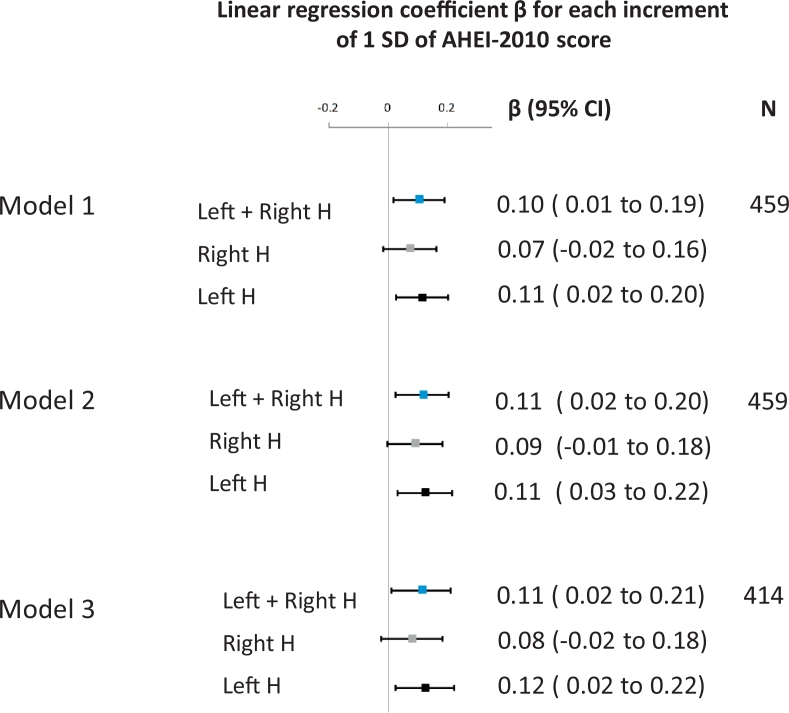
Association between cumulative average of Alternative Healthy Eating Index 2010 over 11-year exposure period (1991–1993–2002–2004) and hippocampal volumes. M1: Model adjusted for age, sex, and total energy intake. M2: M1+ occupational grade, ethnicity, smoking habits, physical activity, cardiometabolic factors, including body mass index, antecedent of coronary heart diseases, hypertension, type II diabetes, and dyslipidemia. M3: M2 + depressive symptoms and cognitive deficit. Hippocampal volumes were normalized using the formula Voladj = vol – b × (intracranial volume – mean intracranial volume ), where b is the regression coefficient of hippocampal volume on intracranial volume, and subsequently scaled to standard deviation units by computing *z* score.

Linear regression models were performed to estimate the association between long-term dietary intake assessed by the cumulative average of AHEI-2010 scores over the exposure period of 11 years (between 1991-1993 and 2002-2004) and normalized hippocampal volumes assessed 13 years later (2015-2016). After adjustment for age, sex, and total energy intake, higher AHEI-2010 score was found to be significantly associated with larger hippocampal volumes (Figure 1). Further adjustment for occupational grade, physical activity, smoking status, and cardiometabolic disorders (model 2), cognitive impairment and depressive symptoms (model 3) confirmed the significant association between higher AHEI-2010 scores and larger hippocampal volume (Figure 1). Each increment of 1 standard deviation of AHEI was associated with an increase of 90.1 mm^3^ (SE = 36.7 mm^3^) and 92.5 mm^3^ (SE = 42.0 mm^3^) larger hippocampal volume for models 2 and 3, respectively.

We further assessed the association between the AHEI-2010 score and hippocampal volume by considering separately the 2 hemispheres and showed that the association was more pronounced in the left hemisphere than in the right one (Figure 1). In the full adjusted model, each increment of 1 standard deviation in AHEI-2010 score was associated with an increase of 56.3 mm^3^ (SE = 23.0 mm^3^) in left hippocampal volume vs 36.2 mm^3^ (SE = 22.7 mm^3^) in right hippocampal volume.

Cardiometabolic disorders, cognitive impairment, and depressive symptoms were considered as potential confounders in the main analyses, but they can also be viewed as potential mediators of the diet-hippocampal volume relationship. In sensitivity analyses excluding participants with cardiometabolic disorder, cognitive impairment, or depressive symptoms, the associations between AHEI and hippocampal volume did not materially differ from those in the main analysis (Supplementary Table 2, available online), making it unlikely that the results are attributable to these conditions.

We further assessed the association between change in AHEI-2010 score over the 11-year exposure period and hippocampal volume. Participants who improved their diet or maintained a high AHEI-2010 score had larger hippocampal volume compared with those who had a low AHEI 2010 score over the exposure period (Supplementary Table 3, available online).

### Dietary Components and Hippocampal Volume

**Figure 2:**
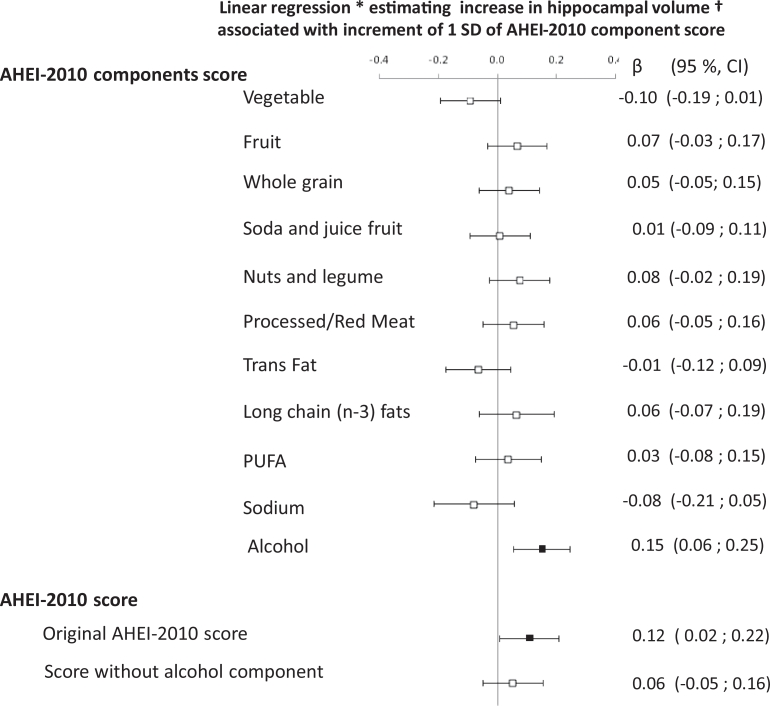
Association between Alternative Healthy Eating Index 2010 (AHEI-2010) component scores and hippocampal volumes. Separate linear regression models were performed, in which each cumulative average of AHEI-2010 component score was included. All component AHEI-2010 scores were standardized by using *z*-scores (mean = 0, standard deviation = 1). Models were adjusted for age, sex, total energy intake, occupational grade, ethnicity, smoking habits, physical activity, cardiometabolic factors, including body mass index, antecedent of coronary heart diseases, hypertension, type II diabetes, dyslipidemia, depressive symptoms, and cognitive deficit. Hippocampal volume was normalized using the formula Voladj = vol – b × (intracranial volume – mean intracranial volume), where b is the regression coefficient of hippocampal volume on ICV and subsequently scaled to standard deviation units by computing the *z*-score. *P* < .05 *P* ≥ .05.

**Supplementary Figure 2:**
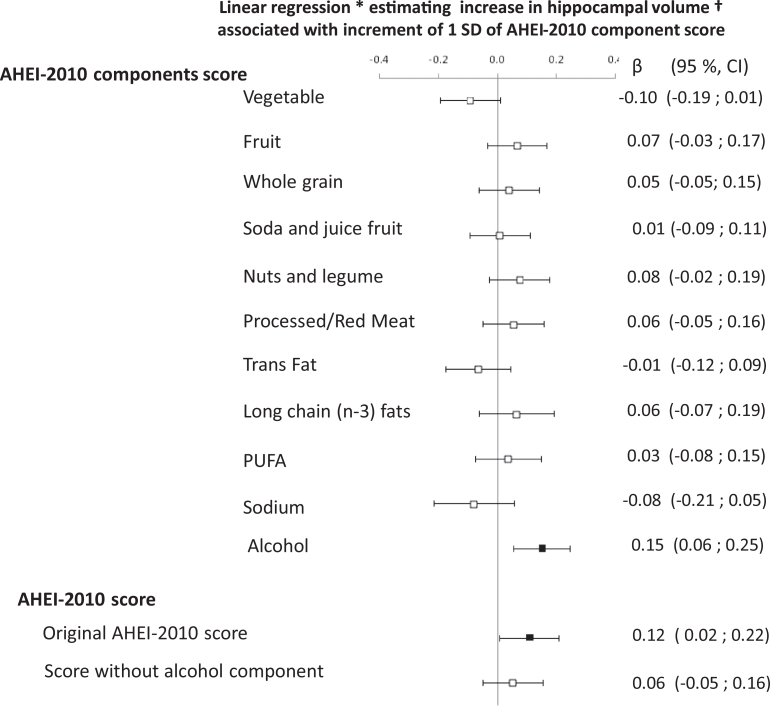
Association between modified AHEI-2010 scores and hippocampal volume.

We further examined which of the 11 components of the AHEI-2010 were most strongly associated with hippocampal volume. Linear regression models were performed to examine the association of cumulative average score of each AHEI-2010 component with total and lateral hippocampal volumes. In an analysis adjusted for sex, age, and total energy intake (Table 3), alcohol consumption was associated with larger hippocampal volumes (total, right, and left), and fruit and red and processed meat components were associated with left hippocampal volume. Only the association between the alcohol component and hippocampal volume persisted in fully adjusted models (Figure 2). The substantial attenuation of the association between the modified AHEI-2010 scores computed without the alcohol component and hippocampal volume suggests that other components contributed little to the association (Figure 2 and Supplementary Figure 2 [available online]).

**Table 3:** Association of Components of AHEI-2010 with Hippocampal Volume

Hippocampal Volume									
AHEI-2010 components*	Total			Right			Left		
Score	Beta	95 % CI	*P*	Beta	95 % CI	*P*	Beta	95 % CI	*P*
Vegetables	−0.05	−0.14 to 0.04	.32	−0.06	−0.14 to 0.04	.30	−0.04	−0.13 to 0.06	.43
Fruits	0.09	0.0001 to 0.18	.05	0.06	−0.03 to 0.15	.23	0.11	0.02 to 0.20	.02
Whole grains	0.05	−0.04 to 0.14	.30	0.04	−0.05 to 0.14	.37	0.05	−0.05 to 0.14	.31
Soda and fruit juice	0.01	−0.08 to 0.10	.80	0.04	−0.06 to 0.13	.43	−0.01	−0.11 to 0.08	.77
Nuts and legumes	0.05	−0.03 to 0.14	.33	0.05	−0.04 to 0.14	.28	0.03	−0.06 to 0.13	.49
Red and processed meat	0.06	−0.03 to 0.16	.17	0.02	−0.08 to 0.11	.70	0.10	0.005 to 0.19	.04
Trans fat	0.02	−0.08 to 0.12	.69	0.003	−0.10 to 0.11	.95	0.03	−0.07 to 0.14	.51
Long-chain (n-3) fats	0.03	−0.09 to 0.14	.53	0.05	−0.06 to 0.17	.29	−0.01	−0.12 to 0.11	.91
Polyunsaturated fatty acids	0.02	−0.09 to 0.12	.77	0.02	−0.08 to 0.13	.68	0.01	−0.10 to 0.11	.90
Sodium	−0.05	−0.18 to 0.07	.39	−0.08	−0.21 to 0.04	.19	−0.02	−0.14 to 0.11	.79
Alcohol	0.15	0.06 to 0.23	.001	0.12	0.03 to 0.21	.01	0.15	0.07 to 0.24	.001

CI = confidence interval.

⁎ Separate linear regression models adjusted for age, sex, and total energy intake with standardized cumulative average of Alternative Healthy Eating Index 2010 component score over the 11-year exposure period as independent variable.

## Discussion

This large observational cohort study examined whether high long-term adherence to dietary guidelines, as assessed with the AHEI-2010 during middle age, was associated with hippocampal volumes 13 years later. Higher cumulative average AHEI-2010 score (reflecting healthy diet) aggregated across repeated measurements was linked to a larger hippocampal volume. This specific association was found to be independent of sociodemographic factors, smoking habits, physical activity, cardiometabolic health factors, cognitive impairment, and depressive symptoms. We further identified low alcohol intake as the key component of AHEI-2010 score independently associated with larger hippocampal volume.

Very few studies have examined whether overall diet is associated with MRI biomarkers in nonclinical study populations. In most of these studies diet quality was assessed by Mediterranean diet score, and higher scores (ie, healthier diet) were found to be associated with larger cortical thickness,^22^, ^23^, ^24^ lower white matter hyperintensity burden,^25^ and preserved white matter microstructure.^26^ Two studies examined the association between adherence to Mediterranean diet and total brain volumes and provided inconclusive answers. In the first study, carried out on 194 elderly adults living in Sweden, no association was found^27^ while in a study of 674 elderly, multi-ethnic, urban-dwelling residents of North Manhattan, New York, high adherence to the Mediterranean diet was associated with larger total brain volume.^22^ In the present study, we report a specific association between healthy diet (assessed by good adherence to AHEI-2010 recommendations) and hippocampal volumes, with a stronger association in left hippocampal volume than in right hippocampal volume. Our findings are in accordance with results from a recent study^11^ in which associations between dietary patterns and hippocampal volumes were assessed in a cohort of 255 Australian older adults. Very similar to our observations, each standard deviation increment in healthy “prudent” dietary pattern (characterized by the consumption of fresh vegetables, fruit, and grilled fish) was found to be associated with a 45.7 mm^3^ larger left hippocampal volume.

Our findings support the hypothesis that a healthy diet may afford protection to the brain by reinforcing hippocampus structures and functions.^28^ This hypothesis was originally formulated based on experimental animal models that suggested a high-energy diet rich in saturated fats and refined sugars adversely affect neuronal plasticity and function. Animals maintained on a high-energy diet rich in fat and sugar showed lower performances in hippocampus-dependent spatial learning,^6^, ^29^^,^^30^ object recognition,^31^ reduced hippocampus levels of brain-derived neurotrophic factor,^30^ impaired in blood-brain barrier integrity^7^ and increase the hippocampal neurogenesis.^32^

The finding that diet-hippocampus volume association was stronger in the left hippocampus than in the right hippocampus remains an intriguing observation. This specific lateral effect of diet on the brain was also reported in other studies.^11^, ^23^ A meta-analysis designed to evaluate the asymmetry of hippocampal volume in control patients with mild cognitive impairment and Alzheimer disease showed a consistent left-smaller-than-right asymmetrical pattern.^33^ However, the underlying mechanisms for this hippocampus asymmetry are largely unknown. Although consistent with other studies, we cannot exclude that this lateral-specific effect of diet on brain structure stems from chance finding.

Low alcohol intake was independently associated with larger hippocampal volumes. This result suggests that the diet-hippocampus structure association was shaped primarily by this component. Our findings corroborate previous findings on Whitehall II demonstrating that alcohol consumption is associated with adverse brain outcomes.^15^ These findings are in line with the literature showing the major deleterious impact of binge drinking and regular intensive drinking on brain^34^, ^35^ and suggest that no or low consumption alcohol intake behavior, compared with high regular alcohol intake, is beneficial in terms of hippocampal volume.

The main strength of this study is the use of a large population-based sample whose participants were administered a comprehensive dietary assessment and who underwent a structural MRI examination 13 years later to acquire detailed data on brain structure. Dietary data were collected using a semi-quantitative FFQ. This method is less precise than those based on weighted records, but it nevertheless covers a range of specific foods and is feasible for large-scale cohort studies such as ours. The validity of FFQs has been criticized^36^ but appears to be reasonable in assessing associations of nutrients and food consumption with outcomes, at least in the UK context.^37^, ^38^ We have shown, for example, that nutrient intakes estimated by the FFQ method are correlated with biomarker concentrations and intake estimates from the 7-day diary. Although the FFQ is open to measurement errors common to all self-reported dietary assessments,^39^ it remains one of the main methods in analytical epidemiological studies.^36^ Indeed, many of the current dietary recommendations and policies to reduce disease burden (eg, obesity, type II diabetes, and cardiovascular disease) rely on evidence from studies using an FFQ.^40^^,^^41^ We assessed healthy diet with AHEI-2010 score, which is based on a set of specific and limited food groups. The measure is assumed to cover all aspects of a “healthy” diet although it may not be adapted to the dietary habits of all populations. The previous findings from the Whitehall II study suggesting that high adherence to AHEI or AHEI-2010 is associated with reduced risk of all-cause and cardiovascular mortality,^42^^,^^43^ long-term inflammation,^44^ and reduced odds of subsequent recurrent depressive symptoms,^45^ support the relevance of using AHEI in the present analysis. Although the dietary assessment preceded brain imaging by several years, and despite adjustment for cognitive impairments and depressive symptoms at the time of the dietary exposure, we cannot exclude the possibility of reverse causation and therefore we are unable to conclude the direction of the association between healthy diet and larger left hippocampal volume. Lastly, we adjusted analyses for many potential confounders and mediators, but with an epidemiological observational framework, our observations may still be explained partly by unmeasured factors, such as cognitive reserve during childhood and adulthood. Further research is also needed to identify mechanisms underlying the observed associations of diet and brain structure, such as changes in metabolic, inflammation, and vascular systems.

In conclusion, our findings lend support for the hypothesis that overall diet may affect brain structures with a specific impact on hippocampal volume. Accounting for the importance of the hippocampus in long-term, declarative, episodic memory, and for flexible cognition network, our findings reaffirm the need to recognize diet and nutrition as potential determinants of cognition, mental health, and social behavior.

## Figures and Tables

**Supplementary Table 1:** Construction of AHEI- 2010 Scores in 464 Participants of the Whitehall II Brain Imaging Substudy in 2002/04

**Components**		**Criteria formin.scores**	**Criteria formax. scores**	**Cumulative averageof AHEI-2010 scoreover 11-y exposureperiod (1991/93-2002/04)**	
				Mean (sd)	Median
Vegetable (serving/day)		0	≥5	5.6 (2.1)	5.7
Fruit (serving/day)		0	≥4	5.7 (2.7)	5.7
Whole grains (serving/day)	Men	0	5	5.5 (2.2)	5.5
	Women	0	6		
Soda and fruit juice (serving/day)		**≥**1	0	3.4 (3.0)	2.7
Nuts and legumes (serving/day)		0	1	4.9 (2.6)	5.0
Processed/Red Meat		**≥**1.5	0	4.6 (2.5)	4.7
Trans Fat (% of energy )		Highest decile	Lowest decile	4.8 (2.6)	4.7
Long-chain (n-3) fats, *mg/d*		0	250	7.9 (2.3)	8.7
PUFA*, *% of energy*		≤2	≥10	5.0 (2.5)	5.0
Sodium, *mg/d*		Highest decile	Lowest decile	4.9 (2.5)	5.0
Alcohol serving/day	Men	**≥**3.5	<1.5	7.5 (3.3)	9.7
	Women	**≥**2.5	<1.0		
Total Score				60.0 (9.0)	59.7

⁎ PUFA (Polyunsaturated fatty acids) does not include n-3 PUFA. Each AHEI component contributed from 0 to 10 points to the total AHEI-2010 score. A score of 10 indicates that the recommendations were fully met, whereas a score of 0 represents the least healthy dietary behavior. Intermediate intakes were scored proportionately between 0 and 10. All the component scores are summed to obtain the total AHEI-2010 score

**Supplementary Table 2:** Associations Between AHEI-2010 Z-Score and Total Hippocampal Volume after Excluding Participants with Cardiometabolic Disorders, Cognitive impairment, and Depressive Symptoms

		Results of linear regression estimating total hippocampus volume* increase per each increment of 1 SD of AHEI-2010 score		
	N analyses	β†	SE	95% IC
Excluding participants with:				
CHD	399	0.11	0.05	0.006 ; 0.21
Type 2 diabete	382	0.15	0.05	0.05 ; 0.26
HTA	295	0.10	0.06	−0.02 ; 0.23
BMI ≥30	345	0.13	0.06	0.02 ; 0.24
Dyslipidemia	345	0.11	0.06	0.0005 ; 0.23
Depressive symptoms	351	0.14	0.05	0.04 ; 0.25
Cognitive impairment	374	0.13	0.05	0.02 ; 0.23

⁎ Hippocampal volumes were normalized using the formula Voladj = vol – b × (ICV – mean ICV). where b is the regression coefficient of hippocampal volumes on ICV. and subsequently scaled to SD units by computing z-score.

† Linear regression models were adjusted for sex. age. total energy intake. occupational grade. ethnicity. smoking status. physical activity and health status factors listed in the table.

**Supplementary Table 3:** Association Between 11-year Change in AHEI-2010 Score and Hippocampal Volume

	Total Hippocampal volume			Right hippocampal volume		Left hippocampal volume	
**10-y change category in AHEI**	n	Beta	95% CI	Beta	95% CI	Beta	95% CI
Maintaining a high AHEI score(Phases 3 and 7 scores ≥ 60.0)	151	0.18	−0.04 ; 0.40	0.14	−0.08 ; 0.36	0.19	−0.04 ; 0.41
vs. low score (Phase 7 andPhase 3 scores < 60.0)	140	ref		ref		ref	
Improving AHEI score (Phase 3score<60.0 andPhase 7 score≥60.0)	75	0.13	− 0.16 ; 0.42	0.04	−0.25 ; 0.32	0.20	− 0.09 ; 0.49
vs. maintaining low score	140	ref		ref		ref	
Decreasing AHEI score (Phase 3score≥60.0 andPhase 7 score<60.0)	80	−0.06	−0.29 ; 0.18	− 0.03	−0.27 ; 0.22	− 0.07	−0.32 ; 0.17
vs. maintaining high score	151	ref		ref		ref	
Maintaining a high AHEI scoreor improving AHEI score	226	0.17	−0.03 ; 0.37	0.11	−0.09 ; 0.31	0.20	−0.005 ; 0.40
vs. low score (Phase 7 andPhase 3 scores<60.0 )	140	ref		ref		Ref	

To analyze the 10-y change in AHEI score, scores of AHEI at phases 3 and 7 were categorized as high or low according to the median value of AHEI-2010 score at phase 3 equal to 60 points. Four categories in 10-y change of AHEI-2010 were then defined: participants who maintained a high score (Phase 3 and 7 scores ≥60.0), those who maintained a low score over the 10-y exposure period (Phase 3 and 7 scores <60.0), participants who improved their AHEI score (Phase 3 score <60.0 and Phase 7 score ≥60.0) and those who decreased their score (Phase 3 score ≥60.0 points and Phase 7 score<60.0 points).

Separate linear regression models adjusted for age, sex and total energy intake differences between phase 7 and phase 3 were performed, in which each category of 10-y change of AHEI-2010 was included. Hippocampal volumes were normalized using the formula Voladj = vol – b × (ICV – mean ICV), where b is the regression coefficient of hippocampal volume on ICV, and subsequently scaled to SD units by computing z-score.

## Supplementary materials

For each of the 127 FFQ items, the selected frequency category was converted to a daily intake. Nutrient intakes were computed by multiplying the consumption frequency for each food by its nutrient content (for specified portions) and then summing nutrient contributions from all foods. Frequency of consumption for multivitamin supplements was also collected. Nutrient values were calculated using the computerized system developed for the Whitehall II dietary data and based on the 4th and 5th editions of McCance and Widdowson's The Composition of Foods and supplementary tables ^1-10^. Nutrient supplement information was obtained from manufacturers of the supplements and added to the database. The validity and reliability of this FFQ in terms of nutrient and food consumption have been documented in detail elsewhere ^11^.

• Chan W, Brown J, Buss D. Miscellaneous Foods. Fourth Supplement to the 5th Edition of McCance and Widdowson's The Composition of Foods. Cambridge; 1994.
• Chan W, Brown J, Lee S. Meat, Poultry and Game. Supplement to the 5th Edition of McCance and Widdowson's The Composition of Foods. Cambridge; 1995.
• Holland B, Brown J, Buss D. Fish and Fish Products: Third Supplement to the 5th Edition of McCance and Widdowson's The Composition of Foods. Cambridge; 1993.
• Holland B, Unwin I, Buss D. Cereals and Cereal product: Third Supplement to Mc Canceand Widdowson's The Composition of Foods. Nottingham; 1988.
• Holland B, Unwin I, Buss D. Milk and Milk Products: Fourth Supplement to Mc Canceand Widdowson's The Composition of Foods. Cambridge; 1989.
• Holland B, Welch A, Buss D. Vegetables, Herbs and Spices: Fifth Supplement to the 4th Edition of McCance and Widdowson's The Composition of Foods. Cambridge; 1991.
• Holland B, Welch A, Buss D. Fruit and Nuts. First Supplement to the 5th Edition of McCance and Widdowson's The Composition of Foods. Cambridge; 1992.
• Holland B, Welch A, Buss D. Vegetable Dishes. Second Supplement to the 5th Edition of McCance and Widdowson's The Composition of Foods. Cambridge; 1992.
• Holland B, Welch A, Unwin I, Buss D, Paul A, Southgate D. McCance and Widdowson's The Composition of Foods. Cambridge; 1991.
• Paul A, Southgate D. McCance and Widdowson's The composition of Foods. 4th Edition ed. London; 1978.
• Bingham SA, Gill C, Welch A et al. Validation of dietary assessment methods in the UK arm of EPIC using weighed records, and 24-hour urinary nitrogen and potassium and serum vitamin C and carotenoids as biomarkers *Int J Epidemiol* 1997;26 Suppl 1:S137-51.

## References

[1] Fleming RM (2000). The pathogenesis of vascular disease. In: Chang JB, ed.*Textbook of Angiologyi*.

[2] Freeman LR, Haley-Zitlin V, Rosenberger DS, Granholm AC (2014). Damaging effects of a high-fat diet to the brain and cognition: a review of proposed mechanisms. Nutr Neurosci..

[3] Lai JS, Hiles S, Bisquera A, Hure AJ, McEvoy M, Attia J (2014). A systematic review and meta-analysis of dietary patterns and depression in community-dwelling adults. Am J Clin Nutr..

[4] Psaltopoulou T, Sergentanis TN, Panagiotakos DB, Sergentanis IN, Kosti R, Scarmeas N (2013). Mediterranean diet, stroke, cognitive impairment, and depression: a meta-analysis. Ann Neurol..

[5] Granholm AC, Bimonte-Nelson HA, Moore AB, Nelson ME, Freeman LR, Sambamurti K (2008). Effects of a saturated fat and high cholesterol diet on memory and hippocampal morphology in the middle-aged rat. J Alzheimers Dis..

[6] Stranahan AM, Norman ED, Lee K (2008). Diet-induced insulin resistance impairs hippocampal synaptic plasticity and cognition in middle-aged rats. Hippocampus..

[7] Kanoski SE, Zhang Y, Zheng W, Davidson TL (2010). The effects of a high-energy diet on hippocampal function and blood-brain barrier integrity in the rat. J Alzheimers Dis..

[8] Ebmeier KP, Donaghey C, Steele JD (2006). Recent developments and current controversies in depression. Lancet..

[9] Sapolsky RM (2001). Depression, antidepressants, and the shrinking hippocampus. Proc Natl Acad Sci U S A..

[10] Schuff N, Woerner N, Boreta L (2009). MRI of hippocampal volume loss in early Alzheimer's disease in relation to ApoE genotype and biomarkers. *Brain.*.

[11] Jacka FN, Cherbuin N, Anstey KJ, Sachdev P, Butterworth P (2015). Western diet is associated with a smaller hippocampus: a longitudinal investigation. BMC Med..

[12] Chiuve SE, Fung TT, Rimm EB (2012). Alternative dietary indices both strongly predict risk of chronic disease. J Nutr..

[13] Filippini N, Zsoldos E, Haapakoski R (2014). Study protocol: the Whitehall II imaging sub-study. BMC Psychiatry..

[14] Marmot M, Brunner E (2005). Cohort profile: the Whitehall II study. Int J Epidemiol..

[15] Topiwala A, Allan CL, Valkanova V (2017). Moderate alcohol consumption as risk factor for adverse brain outcomes and cognitive decline: longitudinal cohort study. BMJ..

[16] Opie RS, Itsiopoulos C, Parletta N (2015;20(3):161-171). Dietary recommendations for the prevention of depression. Nutr Neurosci..

[17] Patenaude B, Smith SM, Kennedy DN, Jenkinson M (2011). A Bayesian model of shape and appearance for subcortical brain segmentation. Neuroimage..

[18] Voevodskaya O, Simmons A, Nordenskjold R (2014). The effects of intracranial volume adjustment approaches on multiple regional MRI volumes in healthy aging and Alzheimer's disease. Front Aging Neurosci..

[19] Sabia S, Dugravot A, Kivimaki M, Brunner E, Shipley MJ, Singh-Manoux A (2012). Effect of intensity and type of physical activity on mortality: results from the Whitehall II cohort study. Am J Public Health..

[20] Crum RM, Anthony JC, Bassett SS, Folstein MF (1993). Population-based norms for the Mini-Mental State Examination by age and educational level. JAMA..

[21] Radloff L (1977). The CES-D scale: a self-report depression scale for research in the general population. Appl Psychol Meas..

[22] Gu Y, Brickman AM, Stern Y (2015). Mediterranean diet and brain structure in a multiethnic elderly cohort. Neurology..

[23] Mosconi L, Murray J, Tsui WH (2014). Mediterranean diet and magnetic resonance imaging-assessed brain atrophy in cognitively normal individuals at risk for Alzheimer's disease. J Prev Alzheimers Dis..

[24] Staubo SC, Aakre JA, Vemuri P (2017;13(2):168-177). Mediterranean diet, micronutrients and macronutrients, and MRI measures of cortical thickness. *Alzheimers Dement.*.

[25] Gardener H, Scarmeas N, Gu Y (2012). Mediterranean diet and white matter hyperintensity volume in the Northern Manhattan Study. Arch Neurol..

[26] Pelletier A, Barul C, Feart C (2015). Mediterranean diet and preserved brain structural connectivity in older subjects. Alzheimers Dementia..

[27] Titova OE, Ax E, Brooks SJ (2013). Mediterranean diet habits in older individuals: associations with cognitive functioning and brain volumes. Exp Gerontol..

[28] Kanoski SE, Davidson TL (2011). Western diet consumption and cognitive impairment: links to hippocampal dysfunction and obesity. Physiol Behav..

[29] Beilharz JE, Maniam J, Morris MJ (2014). Short exposure to a diet rich in both fat and sugar or sugar alone impairs place, but not object recognition memory in rats. Brain Behav Immunity..

[30] Molteni R, Barnard RJ, Ying Z, Roberts CK, Gomez-Pinilla F (2002). A high-fat, refined sugar diet reduces hippocampal brain-derived neurotrophic factor, neuronal plasticity, and learning. Neuroscience..

[31] Heyward FD, Walton RG, Carle MS, Coleman MA, Garvey WT, Sweatt JD (2012). Adult mice maintained on a high-fat diet exhibit object location memory deficits and reduced hippocampal SIRT1 gene expression. Neurobiol Learn Mem..

[32] Stangl D, Thuret S (2009). Impact of diet on adult hippocampal neurogenesis. Genes Nutr..

[33] Shi F, Liu B, Zhou Y, Yu C, Jiang T (2009). Hippocampal volume and asymmetry in mild cognitive impairment and Alzheimer's disease: meta-analyses of MRI studies. Hippocampus..

[34] Agartz I, Momenan R, Rawlings RR, Kerich MJ, Hommer DW (1999). Hippocampal volume in patients with alcohol dependence. Arch Gen Psychiatry..

[35] Beresford TP, Arciniegas DB, Alfers J (2006). Hippocampus volume loss due to chronic heavy drinking. Alcohol Clin Exp Res..

[36] Hebert JR, Hurley TG, Steck SE (2014). Considering the value of dietary assessment data in informing nutrition-related health policy. Adv Nutr..

[37] Bingham SA, Gill C, Welch A (1997). Validation of dietary assessment methods in the UK arm of EPIC using weighed records, and 24-hour urinary nitrogen and potassium and serum vitamin C and carotenoids as biomarkers. Int J Epidemiol..

[38] Brunner E, Stallone D, Juneja M, Bingham S, Marmot M (2001). Dietary assessment in Whitehall II: comparison of 7 d diet diary and food-frequency questionnaire and validity against biomarkers. Br J Nutr..

[39] Webb D, Leahy MM, Milner JA (2013). Strategies to optimize the impact of nutritional surveys and epidemiological studies. Adv Nutr..

[40] Hu FB (2002). Dietary pattern analysis: a new direction in nutritional epidemiology. Curr Opin Lipidol..

[41] Willett W (2013). *Nutritional Epidemiology*. 3rd ed.

[42] Akbaraly TN, Ferrie JE, Berr C (2011). Alternative Healthy Eating Index and mortality over 18 y of follow-up: results from the Whitehall II cohort. Am J Clin Nutr..

[43] Shivappa N, Hebert JR, Kivimaki M, Akbaraly T (2017). Alternate Healthy Eating Index 2010, Dietary Inflammatory Index and risk of mortality: results from the Whitehall II cohort study and meta-analysis of previous Dietary Inflammatory Index and mortality studies. Br J Nutr..

[44] Akbaraly TN, Shipley MJ, Ferrie JE (2015). Long-term adherence to healthy dietary guidelines and chronic inflammation in the prospective Whitehall II study. Am J Med..

[45] Akbaraly TN, Sabia S, Shipley MJ, Batty GD, Kivimaki M (2013). Adherence to healthy dietary guidelines and future depressive symptoms: evidence for sex differentials in the Whitehall II study. Am J Clin Nutr..

