# Supplementary material for: Association of Long-Term Diet Quality with Hippocampal Volume: Longitudinal Cohort Study
Source: Am J Med. 2018 Nov;131(11):1372–1381.e4. doi: 10.1016/j.amjmed.2018.07.001 (PMC6237674; doi:10.1016/j.amjmed.2018.07.001)
Supplement: Supplementary file 1 [file mmc1.docx]

**Online Appendix Text 1**

For each of the 127 FFQ items, the selected frequency category was converted to a daily intake. Nutrient intakes were computed by multiplying the consumption frequency for each food by its nutrient content (for specified portions) and then summing nutrient contributions from all foods. Frequency of consumption for multivitamin supplements was also collected. Nutrient values were calculated using the computerized system developed for the Whitehall II dietary data and based on the 4th and 5th editions of McCance and Widdowson’s The Composition of Foods and supplementary tables ^1-10^. Nutrient supplement information was obtained from manufacturers of the supplements and added to the database. The validity and reliability of this FFQ in terms of nutrient and food consumption have been documented in detail elsewhere ^11^.

1. Chan W, Brown J, Buss D. Miscellaneous Foods. Fourth Supplement to the 5th Edition of McCance and Widdowson's The Composition of Foods. Cambridge; 1994.

2. Chan W, Brown J, Lee S. Meat, Poultry and Game. Supplement to the 5th Edition of McCance and Widdowson's The Composition of Foods. Cambridge; 1995.

3. Holland B, Brown J, Buss D. Fish and Fish Products: Third Supplement to the 5th Edition of McCance and Widdowson's The Composition of Foods. Cambridge; 1993.

4. Holland B, Unwin I, Buss D. Cereals and Cereal product: Third Supplement to Mc Canceand Widdowson's The Composition of Foods. Nottingham; 1988.

5. Holland B, Unwin I, Buss D. Milk and Milk Products: Fourth Supplement to Mc Canceand Widdowson's The Composition of Foods. Cambridge; 1989.

6. Holland B, Welch A, Buss D. Vegetables, Herbs and Spices: Fifth Supplement to the 4th Edition of McCance and Widdowson's The Composition of Foods. Cambridge; 1991.

7. Holland B, Welch A, Buss D. Fruit and Nuts. First Supplement to the 5th Edition of McCance and Widdowson's The Composition of Foods. Cambridge; 1992.

8. Holland B, Welch A, Buss D. Vegetable Dishes. Second Supplement to the 5th Edition of McCance and Widdowson's The Composition of Foods. Cambridge; 1992.

9. Holland B, Welch A, Unwin I, Buss D, Paul A, Southgate D. McCance and Widdowson's The Composition of Foods. Cambridge; 1991.

10. Paul A, Southgate D. McCance and Widdowson's The composition of Foods. 4th Edition ed. London; 1978.

11. Bingham SA, Gill C, Welch A et al. Validation of dietary assessment methods in the UK arm of EPIC using weighed records, and 24-hour urinary nitrogen and potassium and serum vitamin C and carotenoids as biomarkers. *Int J Epidemiol* 1997;26 Suppl 1:S137-51.
