# Supplementary material for: Association of Long-Term Diet Quality with Hippocampal Volume: Longitudinal Cohort Study
Source: Am J Med. 2018 Nov;131(11):1372–1381.e4. doi: 10.1016/j.amjmed.2018.07.001 (PMC6237674; doi:10.1016/j.amjmed.2018.07.001)
Supplement: Supplementary file 2 [file mmc2.docx]

Online Supplementary Material

Table A : Construction of AHEI- 2010 scores in 464 participants of the Whitehall II Brain Imaging Substudy in 2002/04

| **Components** | | | **Criteria for min. scores** | **Criteria for max. scores** | \| **Cumulative average of AHEI-2010 score over 11-y exposure period (1991/93-2002/04)** \| \| --- \| | |
| --- | --- | --- | --- | --- | --- | --- | --- |
|  | | |  |  | Mean (sd) | Median |
| Vegetable (serving /day) | | | 0 | ≥5 | 5.6 (2.1) | 5.7 |
| Fruit (serving /day) | | | 0 | ≥4 | 5.7 (2.7) | 5.7 |
| Whole grains (serving /day) | Men | | 0 | 5 | 5.5 (2.2) | 5.5 |
|  | Women | | 0 | 6 |  |  |
| Soda and fruit juice (serving /day) | | | **≥**1 | 0 | 3.4 (3.0) | 2.7 |
| Nuts and legumes (serving /day) | | | 0 | 1 | 4.9 (2.6) | 5.0 |
| Processed /Red Meat | | | **≥**1.5 | 0 | 4.6 (2.5) | 4.7 |
| Trans Fat (% of energy ) | | | Highest decile | Lowest decile | 4.8 (2.6) | 4.7 |
| Long-chain (n-3) fats, mg/d | | | 0 | 250 | 7.9 (2.3) | 8.7 |
| PUFA*, % of energy | | | ≤2 | ≥10 | 5.0 (2.5) | 5.0 |
| Sodium, mg/d | | | Highest decile | Lowest decile | 4.9 (2.5) | 5.0 |
| Alcohol serving/day | | Men | **≥**3.5 | <1.5 | 7.5 (3.3) | 9.7 |
|  |  | Women | **≥**2.5 | <1.0 |  |  |
| Total Score | | |  |  | 60.0 (9.0) | 59.7 |

* PUFA (Polyunsaturated fatty acids) does not include n-3 PUFA.

Each AHEI component contributed from 0 to 10 points to the total AHEI-2010 score. A score of 10 indicates that the recommendations were fully met, whereas a score of 0 represents the least healthy dietary behavior. Intermediate intakes were scored proportionately between 0 and 10. All the component scores are summed to obtain the total AHEI-2010 score

Table B: Associations between AHEI-2010 z-score and total hippocampal volume after excluding participants with cardiometabolic disorders. cognitive impairment and depressive symptoms.

|  |  | Results of linear regression estimating total hippocampus volume* increase per each increment of 1 SD of AHEI-2010 score | | | |
| --- | --- | --- | --- | --- | --- |
|  | N analyses | β† | SE | 95% IC | |
| Excluding participants with: |  |  |  |  |  |
| CHD | 399 | 0.11 | 0.05 | 0.006 ; 0.21 | |
| Type 2 diabete | 382 | 0.15 | 0.05 | 0.05 ; 0.26 | |
| HTA | 295 | 0.10 | 0.06 | -0.02 ; 0.23 | |
| BMI ≥30 | 345 | 0.13 | 0.06 | 0.02 ; 0.24 | |
| Dyslipidemia | 345 | 0.11 | 0.06 | 0.0005 ; 0.23 | |
| Depressive symptoms | 351 | 0.14 | 0.05 | 0.04 ; 0.25 | |
| Cognitive impairment | 374 | 0.13 | 0.05 | 0.02 ; 0.23 | |

* Hippocampal volumes were normalized using the formula Voladj = vol – b × (ICV – mean ICV). where b is the regression coefficient of hippocampal volumes on ICV. and subsequently scaled to SD units by computing z-score.

† Linear regression models were adjusted for sex. age. total energy intake. occupational grade. ethnicity. smoking status. physical activity and health status factors listed in the table.

Table C : Association between 11-year change in AHEI-2010 score and hippocampal volume

|  | Total Hippocampal volume | | |  | Right hippocampal volume | |  | Left hippocampal volume | |
| --- | --- | --- | --- | --- | --- | --- | --- | --- | --- |
| **10-y change category in AHEI** | n | Beta | 95% CI |  | Beta | 95% CI |  | Beta | 95% CI |
| Maintaining a high AHEI score (Phases 3 and 7 scores ≥ 60.0) | 151 | 0.18 | -0.04 ; 0.40 |  | 0.14 | -0.08 ; 0.36 |  | 0.19 | -0.04 ; 0.41 |
| vs. low score (Phase 7 and Phase 3 scores < 60.0) | 140 | ref |  |  | ref |  |  | ref |  |
|  |  |  |  |  |  |  |  |  |  |
| Improving AHEI score (Phase 3 score<60.0 and Phase 7 score≥60.0) | 75 | 0.13 | - 0.16 ; 0.42 |  | 0.04 | -0.25 ; 0.32 |  | 0.20 | - 0.09 ; 0.49 |
| vs. maintaining low score | 140 | ref |  |  | ref |  |  | ref |  |
|  |  |  |  |  |  |  |  |  |  |
| Decreasing AHEI score (Phase 3 score≥60.0 and Phase 7 score<60.0) | 80 | -0.06 | -0.29 ; 0.18 |  | - 0.03 | -0.27 ; 0.22 |  | - 0.07 | -0.32 ; 0.17 |
| vs. maintaining high score | 151 | ref |  |  | ref |  |  | ref |  |
|  |  |  |  |  |  |  |  |  |  |
| Maintaining a high AHEI score or improving AHEI score | 226 | 0.17 | -0.03 ; 0.37 |  | 0.11 | -0.09 ; 0.31 |  | 0.20 | -0.005 ; 0.40 |
| vs. low score (Phase 7 and Phase 3 scores<60.0 ) | 140 | ref |  |  | ref |  |  | ref |  |

To analyze the 10-y change in AHEI score, scores of AHEI at phases 3 and 7 were categorized as high or low according to the median value of AHEI-2010 score at phase 3 equal to 60 points. Four categories in 10-y change of AHEI-2010 were then defined: participants who maintained a high score (Phase 3 and 7 scores ≥60.0), those who maintained a low score over the 10-y exposure period (Phase 3 and 7 scores <60.0), participants who improved their AHEI score (Phase 3 score <60.0 and Phase 7 score ≥60.0) and those who decreased their score (Phase 3 score ≥60.0 points and Phase 7 score<60.0 points).

Separate linear regression models adjusted for age, sex and total energy intake differences between phase 7 and phase 3 were performed, in which each category of 10-y change of AHEI-2010 was included. Hippocampal volumes were normalized using the formula Voladj = vol – b × (ICV – mean ICV), where b is the regression coefficient of hippocampal volume on ICV, and subsequently scaled to SD units by computing z-score.
