# Supplementary material for: Association of Long-Term Diet Quality with Hippocampal Volume: Longitudinal Cohort Study
Source: Am J Med. 2018 Nov;131(11):1372–1381.e4. doi: 10.1016/j.amjmed.2018.07.001 (PMC6237674; doi:10.1016/j.amjmed.2018.07.001)
Supplement: Supplementary file 3 [file mmc3.pptx]

## Slide 1
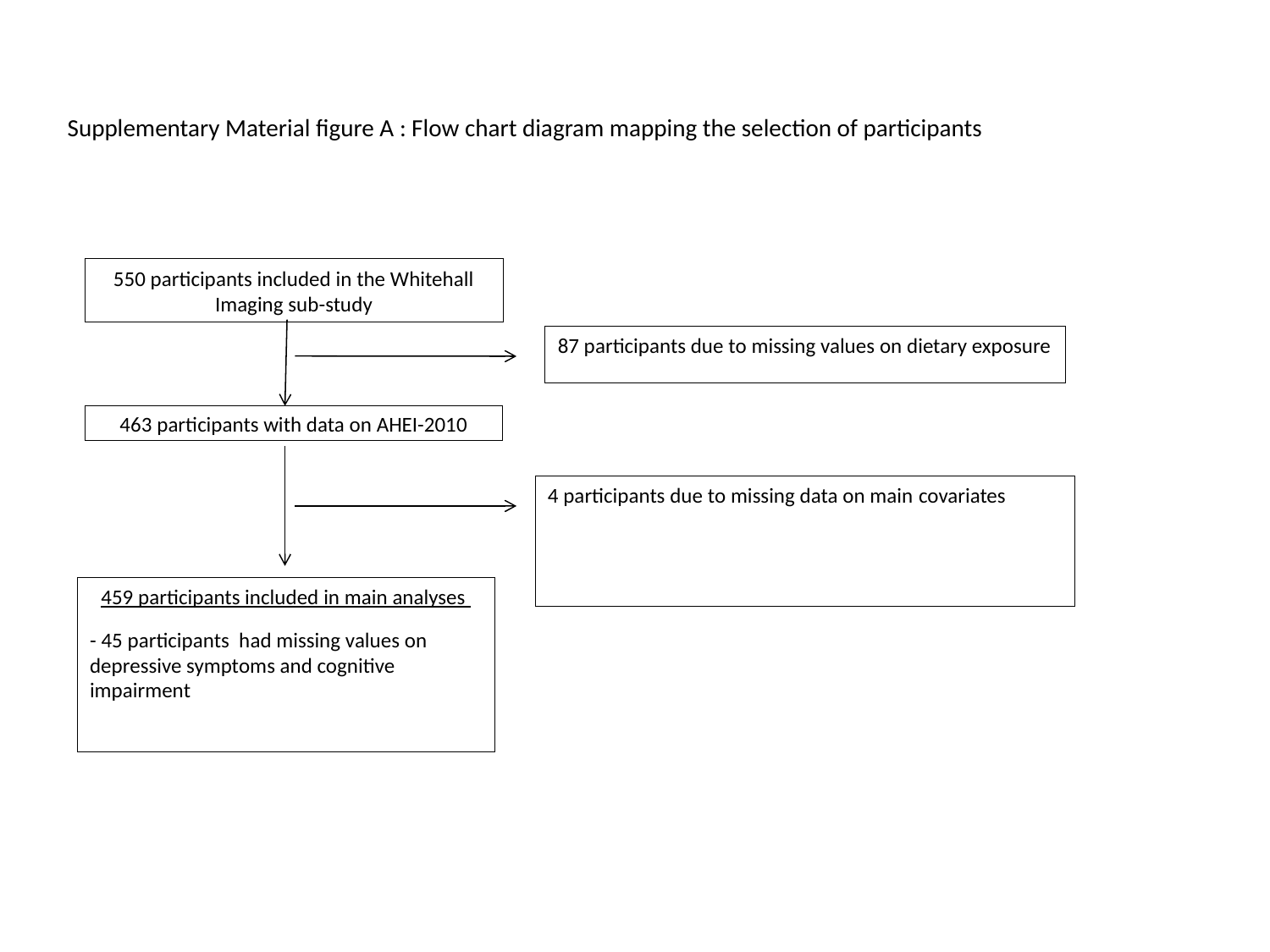

Supplementary Material figure A : Flow chart diagram mapping the selection of participants
550 participants included in the Whitehall Imaging sub-study
87 participants due to missing values on dietary exposure
463 participants with data on AHEI-2010
4 participants due to missing data on main covariates
459 participants included in main analyses
- 45 participants had missing values on depressive symptoms and cognitive impairment
