# Supplementary material for: Association of Long-Term Diet Quality with Hippocampal Volume: Longitudinal Cohort Study
Source: Am J Med. 2018 Nov;131(11):1372–1381.e4. doi: 10.1016/j.amjmed.2018.07.001 (PMC6237674; doi:10.1016/j.amjmed.2018.07.001)
Supplement: Supplementary file 4 [file mmc4.pptx]

## Slide 1
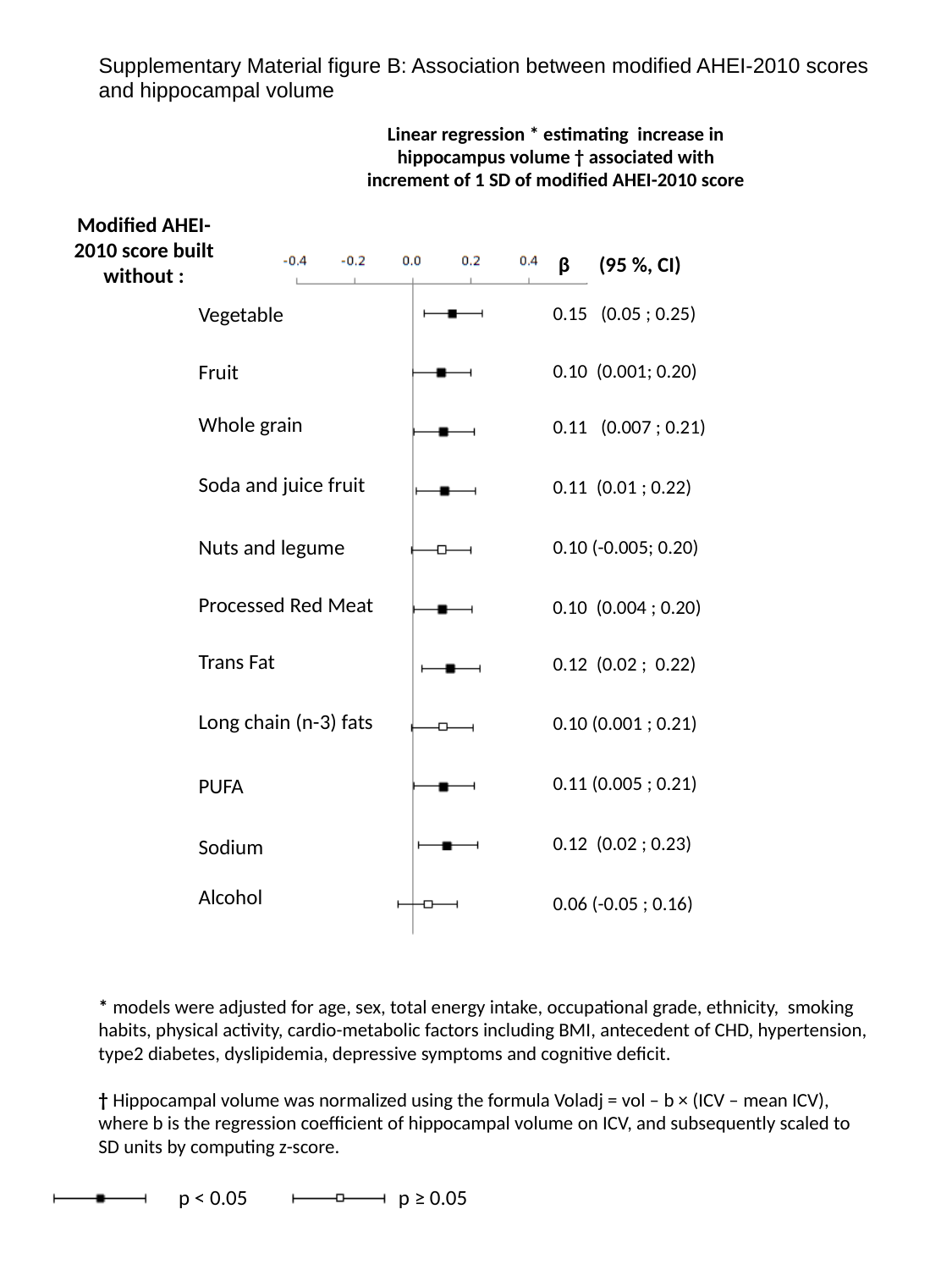

Supplementary Material figure B: Association between modified AHEI-2010 scores and hippocampal volume
Linear regression * estimating increase in hippocampus volume † associated with increment of 1 SD of modified AHEI-2010 score
Modified AHEI-2010 score built
 without :
β (95 %, CI)
Vegetable
Fruit
Whole grain
Soda and juice fruit
Nuts and legume
Processed Red Meat
Trans Fat
Long chain (n-3) fats
PUFA
Sodium
Alcohol
 0.15 (0.05 ; 0.25)
 0.10 (0.001; 0.20)
 0.11 (0.007 ; 0.21)
 0.11 (0.01 ; 0.22)
 0.10 (-0.005; 0.20)
 0.12 (0.02 ; 0.22)
 0.10 (0.001 ; 0.21)
 0.11 (0.005 ; 0.21)
 0.12 (0.02 ; 0.23)
 0.06 (-0.05 ; 0.16)
 0.10 (0.004 ; 0.20)
* models were adjusted for age, sex, total energy intake, occupational grade, ethnicity, smoking habits, physical activity, cardio-metabolic factors including BMI, antecedent of CHD, hypertension, type2 diabetes, dyslipidemia, depressive symptoms and cognitive deficit.
† Hippocampal volume was normalized using the formula Voladj = vol – b × (ICV – mean ICV), where b is the regression coefficient of hippocampal volume on ICV, and subsequently scaled to SD units by computing z-score.
p < 0.05
p ≥ 0.05
